# An Investigation into the Perceptions of Veterinarians towards Calf Welfare in New Zealand

**DOI:** 10.3390/ani11020421

**Published:** 2021-02-06

**Authors:** Ria van Dyke, Amy Miele, Melanie Connor

**Affiliations:** Division of Animal Behaviour and Welfare, The Royal (Dick) School of Veterinary Studies, University of Edinburgh, Roslin, Edinburgh EH25 9RG, UK; ria.vandyke@gmail.com (R.v.D.); m.connor@irri.org (M.C.)

**Keywords:** animal welfare, calves, veterinarians, legislation, calf management, perceptions

## Abstract

**Simple Summary:**

While developments in animal welfare science have led to a greater understanding of the welfare needs of calves (*Bos taurus*), there are prevailing concerns that current knowledge has not been adopted in practice. Given that the perceptions of veterinarians have direct implications for the level of welfare protection afforded to calves, this study investigated the current thinking of veterinarians towards the welfare of young calves in New Zealand. Through a nationwide survey, the findings revealed that veterinarians strongly disagreed with the specifications of certain calf welfare regulations. Veterinarians also expressed diverse concerns for the potential risks of calf welfare compromise across the production chain and identified multiple barriers to implementing welfare-related change. The findings indicate considerable support among veterinarians for strengthening the level of welfare protection afforded to calves in New Zealand. Given the asymmetries that exist between the current regulatory framework and veterinary perspectives, the findings suggest that more needs to be done to improve calf welfare in New Zealand.

**Abstract:**

Despite recent legislative amendments to address areas of highest risk to the welfare of calves (*Bos taurus*) in New Zealand, there are prevailing concerns that animal welfare science knowledge has not been adopted in practice. As a part of a larger, nationwide study investigating the perceptions of veterinarians towards calf welfare, the aim of the current work was to investigate the perceptions of veterinarians towards the level of welfare protection afforded to young “bobby” calves in New Zealand. This study also explored concerns for welfare compromise and identified barriers to welfare-related change for calves more generally. An electronic mixed-methods survey was completed by 104 veterinarians registered with the Veterinary Council of New Zealand. The findings revealed that veterinarians strongly disagreed with the specifications of certain calf welfare regulations. Veterinarians also identified areas at highest risk of calf welfare compromise across the production chain and barriers to welfare-related change. These findings demonstrate considerable support among veterinarians for improving the level of welfare protection afforded to calves. Given the discrepancies that exist between the current regulatory regime and veterinary perspectives, the knowledge gained from this study can be used in support of regulatory reform to strengthen calf welfare in practice and policy in New Zealand.

## 1. Introduction

In recent years, the welfare of calves (*Bos taurus*) has come under increasing scrutiny in New Zealand [1]. In 2019, there were 5.4 million calves born across New Zealand’s dairy and beef industries [2]. Every year, approximately 40% are considered a by-product of the dairy industry, and slaughtered between the ages of 4 and 7 days old [3]. These “bobby” calves are particularly vulnerable due to the very young age at which they are removed from their dam, handled, and transported prior to slaughter [1,4]. Given that welfare inputs are inextricably linked to economic outputs [5,6,7], and the economic value of bobby calves is considered nominal, the low prioritization of young calves raises important concerns for their welfare [7,8,9,10].

In 2015, a high-profile media exposé depicted widespread mistreatment of bobby calves across the production chain in New Zealand [11]. Following intense global scrutiny, the National Animal Welfare Advisory Committee (NAWAC) and the Ministry for Primary Industries (MPI) met with industry stakeholders to identify areas of highest risk to the welfare of young calves [3]. The areas where regulations were considered an appropriate mechanism to address the concerns were developed into regulatory proposals and public submissions were invited in 2016 [3]. Despite concern that the proposed standards would otherwise fall below the general provisions of the Animal Welfare Act 1999 (henceforth “the Act”) [12,13], the regulations were introduced and incorporated in the Animal Welfare (Care and Procedures) Regulations 2018.

The New Zealand Veterinary Association (NZVA) has emphasized the necessity of targeted interventions to improve calf welfare, affirming that the Act must not only protect cattle from overt cruelty, but also protect their physical and psychological health and welfare [14,15]. Given that veterinarians are considered educators [7], advisors [16,17], influencers [7,18,19,20], and informed assessors of animal welfare [21], the perceptions of veterinarians have direct implications for the level of welfare protection afforded to calves in practice [22,23,24].

While veterinarians are uniquely positioned to offer valuable insights into current calf management practices, limited knowledge exists regarding the perceptions of veterinarians towards the welfare needs of calves. Relatively few studies regarding veterinary perspectives on cattle welfare are available in the scientific literature [7,21,22,23,24,25,26]. Of the research available, most focus on attitudes towards pain management [21,22,23,24], with relatively few studies concerning the perspectives of veterinarians towards other welfare considerations of common management practices [7,25]. In particular, there are no published studies examining the perceptions of veterinarians towards the current welfare provisions afforded to calves, and this merits further investigation. Further, very few studies have reported on veterinary perspectives concerning calf welfare compromise and barriers to welfare-related change [7,25]. Understanding the human component of animal management is central to finding solutions that lead to sustained improvements in animal welfare [27]. In order to facilitate the development of targeted strategies aimed at improving the welfare protection afforded to calves, there is a need to identify areas of highest risk, along with barriers that may impact upon such efforts [28].

The objective of this study was to investigate the current thinking among veterinarians regarding calf welfare in New Zealand. More specifically, this study sought: (i) to examine whether the current thinking among veterinarians aligns with the specifications of certain welfare provisions afforded to young “bobby” calves; (ii) to determine whether certain demographic factors influence opinions towards those welfare provisions; (iii) to explore shared concerns for calf welfare compromise; and (iv) to identify perceived barriers to implementing welfare-related change. Given that the current regulatory framework in New Zealand does not differentiate between the dairy or beef industry, a distinction was not made in the current work.

## 2. Materials and Methods

This paper is part of a larger, nationwide study investigating the perceptions of veterinarians towards the level of welfare protection afforded to calves in New Zealand. Described here are the methods involved in exploring veterinary perspectives towards the current welfare provisions for young calves (up to 14 days of age).

### 2.1. Ethical Approval

Prior to commencement, ethical approval was obtained from the University of Edinburgh Human Ethical Committee (HERC_269-18).

### 2.2. Survey Development

Facilitated by the empirical literature, a mixed-methods survey was developed to investigate the perceptions of veterinarians towards calf welfare through open dialogue with veterinarians, academics, and veterinary students. Initial pilot interviews were carried out with a small sample of veterinary students to test the survey for applicability and comprehensibility. The survey was then electronically distributed (Jisc Online Surveys) to a second sample of veterinary students for the purposes of pilot testing.

The first section of the survey focused on collecting quantifiable characteristics of the sample, including: graduation year, gender, birth year, and species emphasis. In order to explore whether veterinary perceptions align with current calf welfare regulations, the survey asked participants to consider three recent regulatory amendments and select the multiple-choice option that best reflected their opinion (Table 1). Participants were given the opportunity to select “other” and provide their own answer if desired.

In order to identify shared concerns regarding the areas at highest risk of calf welfare compromise, participants were then asked to provide up to three concerns for calf welfare compromise in order of concern, with the first response being the most concerning.

To explore veterinary perceptions towards barriers to welfare-related change, participants were also asked to provide up to three perceived barriers to change in ranked order of importance. As a relatively new area of exploratory research, the survey sought to determine whether these qualitative findings corroborated with the quantitative data by asking participants to then rate certain barriers to welfare-related change on a 6-point Likert-type scale (1 = not at all, 6 = definitely). Potential barriers were adapted from the existing literature (Table 2). Participants were provided with the opportunity to elaborate on any other perceived barriers if they desired.

### 2.3. Sampling

Permission to sample veterinarians with a practicing certificate through the Veterinary Council of New Zealand (VCNZ) was granted in late 2018. A list of veterinarians was retrieved from the VCNZ register and compiled into a database (Microsoft Excel 2016), which comprised of 604 veterinarians. Inclusion in the database was based on veterinarians who displayed a direct e-mail contact and were listed on the register as currently working in clinical practice in New Zealand.

Given that the current work was developed in the wake of global scrutiny towards calf welfare in New Zealand and subsequent legislative transformation, safeguarding respondent identity through full anonymization was particularly important. To reduce the perception of personal risk and enhance self-disclosure, the survey enabled anonymous self-administration.

A cover letter introducing the nature of the research, along with a link to the survey (Jisc Online Surveys), was electronically administered via e-mail to all veterinarians included in the database. This was followed two weeks later with a courtesy message thanking those who had responded and appealing to others to respond. This approach follows Dillman’s [37] general recommendations for survey protocol to improve the rate of response.

### 2.4. Data Analysis

The data was exported from Jisc Online Surveys directly into the Statistical Package for the Social Sciences (SPSS Version 24). The level of significance was *p* < 0.05.

Sample demographics were reported as frequencies, along with measures of central tendency and distribution. An independent samples t-test was used to explore differences between gender for continuous variables. Chi-square analysis was used for investigating the association between categorical demographic variables.

Responses to the legislative items were reported as frequencies. Fisher’s exact test was utilized to investigate whether demographic factors influenced perceptions towards certain legislative specifications, given that over 20% of the expected values were <5. Where the effect was found to be significant, post hoc pairwise comparisons were utilized to analyze the direction of effect with Bonferroni correction to reduce Type I errors.

Qualitative ranked responses for calf welfare compromise and barriers to welfare-related change were entered into a database (Microsoft Excel 2016) and coding was independently cross-checked with a second coder in an open process where assumptions could be challenged, and an agreement reached. Through a transformative process, coded responses were then quantified with a weighted rank score (WRS):

$${\mathrm{WRS}}_{c}=3\cdot \sum c_{rank\;1}+2\cdot \sum c_{rank\;2}+1\cdot \sum c_{rank\;3}$$

The statistical Equation was developed to take into account the frequency of the code and its weighted rank to ensure that the greatest concerns had a proportional representation during analysis. For each unique code (*c*), the weighted rank score (WRS*_c_*) was calculated as the sum of the number of occurrences of the unique code at each (*n*th) rank (∑*c_rank n_*), multiplied by the rank’s weight constant. A higher WRS indicated greater concern among veterinarians. Given that it is not possible to quantify the distance between each rank, the equation serves as a guide and is not intended to be interpreted in isolation or to replace the full dataset.

Responses to the Likert-type items concerning barriers to welfare-related change were reported as frequencies, along with median and interquartile range values. Mann–Whitney U tests were used for two sample comparisons, and Kruskal–Wallis H comparisons for multilevel samples. To determine the direction of effect, pairwise Mann–Whitney U tests were performed. Significance levels were subject to Bonferroni correction to reduce the impact of Type I errors. Participants were also given the opportunity to elaborate further and responses were coded according to their content. Of the qualitative responses, those which were most widely shared among veterinarians were reported and an example of each was provided.

## 3. Results

In total, 104 veterinarians were included in the final dataset. Of the 106 surveys returned, one submission with all missing entries, along with another with only demographic data provided, were excluded from the study.

### 3.1. Demographic Data

A breakdown of the sample demographics is included in Table 3. The mean age of the veterinarians that participated in the study was 48.8 (σ = 13.9) years. Female veterinarians (μ = 42.7, σ = 11.6, *n* = 53) were significantly younger than male veterinarians (μ = 55.4, σ = 13.4, *n* = 49; *t* (100) = 5.11, *p* < 0.001). The mean number of years since graduation was 23.8 years (σ = 14.3, *n* = 103). Female veterinarians (μ = 17.3, σ = 11.3, *n* = 52) had graduated more recently than male veterinarians (μ = 30.8, σ = 14.1, *n* = 49; *t* (99) = 5.30, *p* < 0.001). There were no significant differences between the number of male and female participants specializing in a certain species (χ^2^ (2) = 4.53, *p* = 0.104).

### 3.2. Minimum Fitness for Transport (Age)

To investigate whether the perceptions of veterinarians align with current calf welfare legislation, veterinarians provided their opinions on three legislative items concerning young calves (up to 14 days of age). Results from the quantitative data indicated that the majority of veterinarians (58.2%; *n* = 60) supported a higher age of fitness for transport than the current 4 day minimum. Support was highest for increasing the minimum age to 10 days (35.9%; *n* = 37). There were 9 participants who chose to provide their own answer. Some of the answers which were provided included: 24 h, 5 days, 7 days, 21 days, and 4 weeks. Other answers expressed that the age depends on the length of the journey, or disagreed that young calves should be transported at all.

**Figure 1:**
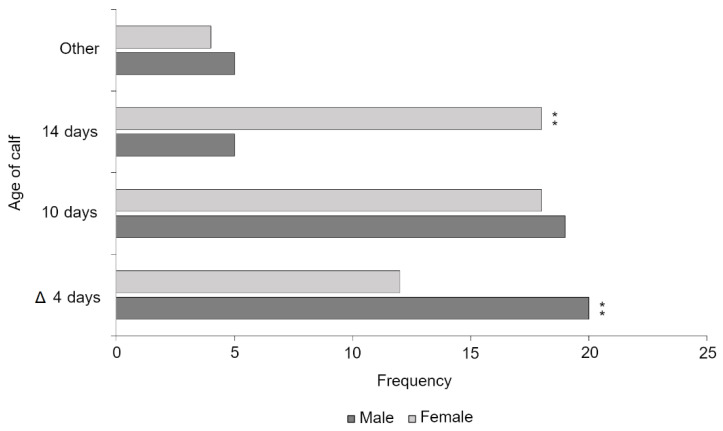
Veterinary opinion on the minimum age at which a young calf is fit for transport. The current legislative minimum age of fitness for transporting young calves has been denoted by a delta symbol. Significant differences between genders following Bonferroni correction (0.05/3 = 0.017) have been marked with a double asterisk (*p* = 0.005).

There was a significant difference in responses between male and female veterinarians (*p* = 0.020). Females (*n* = 49) supported a higher mean age of fitness for transport (μ = 10.41 days; σ = 4.81) than males (*n* = 48; μ = 8.06 days; σ = 4.10). Post hoc pairwise comparisons with Bonferroni correction (0.05/3 = 0.017) demonstrated that female veterinarians were significantly more likely to select a 14 day minimum age of fitness for transport than male veterinarians, who were more likely to select a 4 day minimum age (*p* = 0.005; Figure 1).

**Figure 2:**
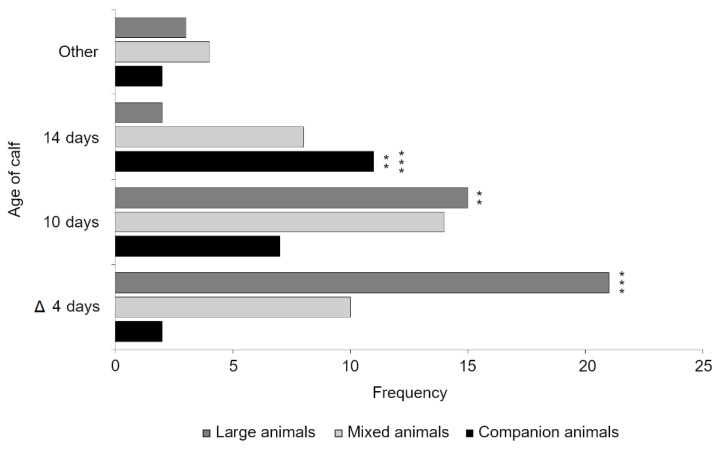
Veterinary opinion on the minimum age at which a young calf is fit for transport. The current legislative minimum age of fitness for transporting young calves has been denoted by a delta symbol. Significant differences between species emphasis following Bonferroni correction (0.05/9 = 0.006) have been marked with a double and triple asterisk (*p* = 0.005 and *p* = 0.001, respectively).

There was also a significant difference between veterinarians working with different species (*p* = 0.001). Veterinarians working in large animal practice (*n* = 39) supported a lower age of fitness for transport (μ = 7.26 days, σ = 4.02) than veterinarians working in companion animal practice (*n* = 21, μ = 11.71 days, σ = 3.18) and mixed animal practice (*n* = 35, μ = 9.54 days, σ = 5.19). Post hoc pairwise comparisons with Bonferroni correction (0.05/9 = 0.006) found that veterinarians working in large animal practice were significantly more likely to select a 4 or 10 day minimum age of fitness for transport than veterinarians working with companion animals, who were more likely to select a 14 day minimum age (*p* = 0.001 and *p* = 0.005, respectively; Figure 2). The number of years since graduation was not found to be significant (*p* = 0.998).

### 3.3. Maximum Time off Feed Prior to Slaughter

Veterinarians widely disagreed with the current 24 h maximum time off feed for young calves prior to slaughter (97.1%; *n* = 101). Most veterinarians answered that 6 or 12 h off feed was an acceptable maximum limit (47.1%; *n* = 49 and 44.2%; *n* = 46, respectively). There were 6 veterinarians who provided their own answer, including 1 h, 2 h, 3 h, and 4h. A single veterinarian expressed the difficulty in monitoring time off feed during long transport journeys.

**Figure 3:**
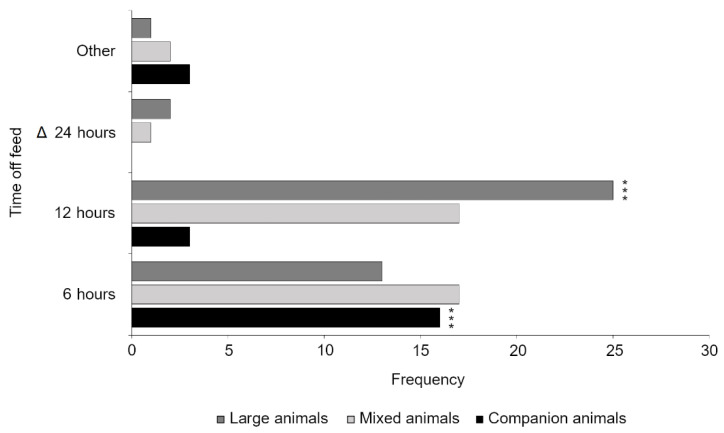
Veterinary opinion on the maximum time off feed prior to slaughter for young calves. The current legislative minimum time off feed for young calves has been denoted by a delta symbol. Significant differences between species emphasis following Bonferroni correction (0.05/9 = 0.006) have been marked with a triple asterisk (*p* = 0.001).

There was a significant difference in the responses between veterinarians working with different species (*p* = 0.003). Veterinarians working with companion animals (*n* = 22) supported a lower time off feed (μ = 6.32 h, σ = 2.68) than veterinarians working in large animal practice (*n* = 40, μ = 10.65 h, σ = 4.19) and mixed animal practice (*n* = 37, μ = 9.04 h, σ = 4.16). More specifically, post hoc pairwise comparisons with Bonferroni correction (0.05/9 = 0.006) found that veterinarians in companion animal practice were significantly more likely to select a 6 h maximum time off feed than veterinarians working in large animal practice, who were instead more likely to select 12 h off feed (*p* = 0.001; Figure 3). Gender (*p* = 0.618) and years since graduation (*p* = 0.571) were not found to be significant.

### 3.4. Maximum Transport Duration

Participants strongly disagreed with the current 12 h maximum duration of transport for young calves (99.0%; *n* = 103). Most participants considered a 6 h maximum limit as acceptable (61.9%; *n* = 65). Of those participants who chose to provide their own answer, the majority specified an answer below 6 h (99%; *n* = 30). A single participant expressed that while the duration would depend on the age of the calf, a 6 h journey would cause stress for a 4 day old calf. Demographic differences between gender (*p* = 0.201), species emphasis (*p* = 0.425), and years since graduation (*p* = 0.190), were not statistically significant.

**Figure 4:**
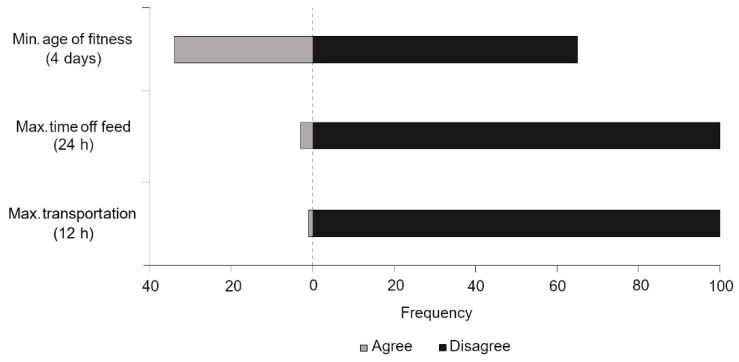
Frequency of veterinarians that agreed or disagreed with certain specifications of calf welfare legislation, where the dashed line represents the point between agreement/disagreement.

In brief, the majority of veterinarians disagreed with the current level of welfare protection across three legislative items: minimum age of fitness for transport (4 days), maximum time off feed prior to slaughter (24 h), and maximum transport duration (12 h). The level of agreement is summarized in Figure 4. For clarity purposes, veterinarians that were unable to form an opinion on a given practice were not included in this graph.

### 3.5. Veterinary Concerns for Calf Welfare Compromise

**Figure 5:**
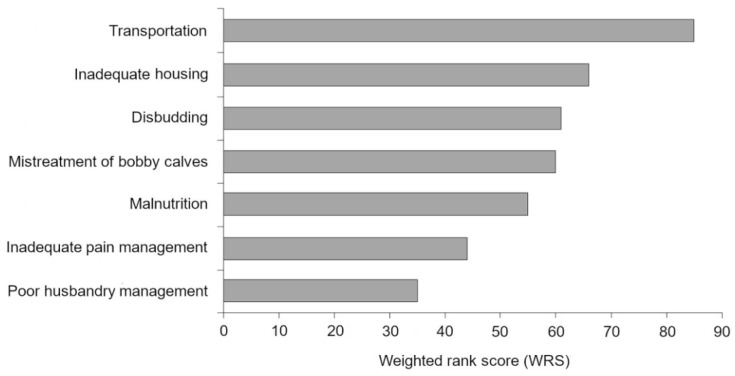
Veterinarians’ concerns on the areas perceived to represent the greatest risk of calf welfare compromise, based on coded qualitative responses. The weighted rank score (WRS) takes into account the frequency of the code and its weighted rank to ensure the greatest concerns had a proportional representation. For each code, the WRS was calculated as the sum of the number of occurrences of the code at each rank, multiplied by the rank’s weight constant. A higher WRS indicates greater concern among veterinarians.

Rank responses for the areas at highest risk of calf welfare compromise underwent qualitative content analysis and were quantified according to their proportional rank. The areas most widely identified as important welfare risks were transportation, inadequate housing, disbudding, and the mistreatment of bobby calves. The results are summarized in Figure 5. Given the broad range of responses, only those comprising at least 5% or more of the sample were included in the graph, as those concerns were more widely shared among the veterinarians (see Table A1 (Appendix A) for a full breakdown).

### 3.6. Perceived Barriers to Welfare-Related Change

**Figure 6:**
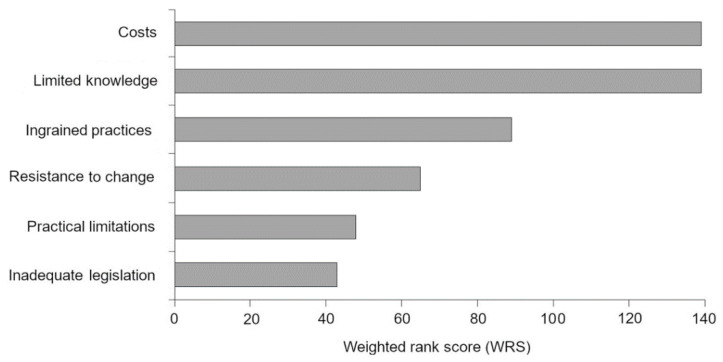
Veterinarians’ perceived barriers to welfare-related change, based on coded qualitative responses. The WRS takes into account the frequency of the code and its weighted rank to ensure the greatest concerns had a proportional representation. For each code, the WRS was calculated as the sum of the number of occurrences of the code at each rank, multiplied by the rank’s weight constant. A higher WRS indicates greater concern among veterinarians.

Veterinarians identified multiple areas of influence that may inhibit welfare-related change. Of those identified, veterinarians perceived costs, limited knowledge, and ingrained practices as the greatest barriers to welfare-related change (Figure 6). Given the broad range of responses, only those comprising at least 5% or more of the sample were included in the graph, as those concerns were more widely shared among the veterinarians (see Table A2 (Appendix B) for a full breakdown of responses).

**Figure 7:**
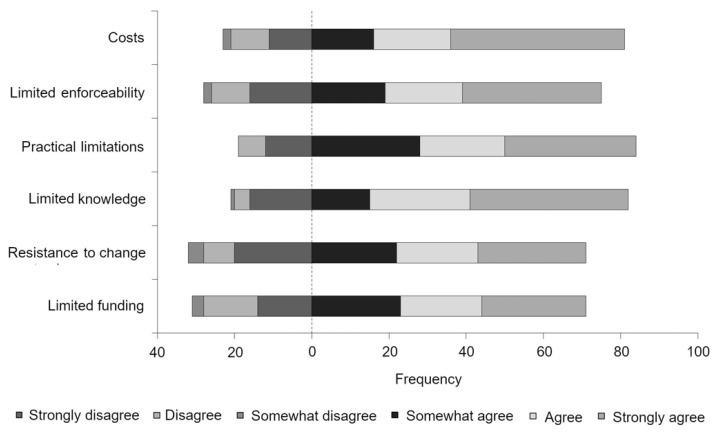
Veterinary perceptions towards barriers to welfare-related change, where the dashed line represents the point between disagreement and agreement based on responses to a 6-point Likert-type scale.

Participants provided their opinions to certain barriers on a 6-point Likert-type scale. All scale items were considered important barriers to welfare-related change (Figure 7). Participants mostly agreed that the following items presented the greatest barriers to welfare-related change: animal welfare knowledge (79.6%), increased costs (77.9%), and practical limitations of monitoring (81.6%; all Med = 5.0; IQR = 4.0–6.0). Veterinarians working in companion animal practice perceived that limited enforceability presented a greater barrier to welfare-related change than veterinarians working in large animal practice (*U*(*N*_COMP_ = 22, *N*_LARGE_ = 41) = 283.50, *z* = −2.51, *p* = 0.012). Further, veterinarians working in companion animal practice were more likely to identify resistance to change as a barrier than veterinarians working in large or mixed animal practice (*U*(*N*_COMP_ = 22, *N*_LARGE_ = 41) = 256.50, *z* = −2.89, *p* = 0.004; *U*(*N*_COMP_ = 22, *N*_MIXED_ = 36) = 241.50, *z* = −2.56, *p* = 0.010, respectively). Gender and the number of years since graduation were not found to have a significant effect on the level of concern for any of the scale items (all *p* > 0.05).

Participants were given the opportunity to elaborate on any additional barriers to welfare-related change. Of the 13 responses, the most representative concerns included a lack of empathy, “calves are not viewed as living beings […]”; limited knowledge, “it needs to be a change made […] after being educated and coming to the conclusions themselves that it is the best thing to do”; and practical limitations “[…] for the farmers in their busiest time of year”. Concern was also expressed for navigating veterinary ethics in practice, “there is often a reluctance to take a very strong position on welfare issues [...] because we need to protect a client relationship that brings business. How do you take a strong position on welfare and still have clients welcome you onto farm?”

## 4. Discussion

The aim of this study was to investigate the perceptions of veterinarians towards calf welfare in New Zealand. This research offered an opportunity for veterinarians to respond to the recent legislative changes and voice their opinion on calf welfare in New Zealand under the condition of anonymity, without personal or social repercussions of disclosure. This study serves as an effort to investigate the current thinking of veterinarians towards calf welfare legislation, areas at highest risk of calf welfare compromise, and barriers to welfare-related change. The results found that veterinarians strongly disagreed with the specifications of certain calf welfare regulations, shared a broad range of concerns regarding the potential for calf welfare compromise across the production chain, and identified multiple barriers to welfare-related change.

### 4.1. Veterinary Perceptions towards Calf Welfare Legislation

Counter to the current 4 day minimum age of fitness for transport, the majority of veterinarians did not support the transport of young calves under 10-days of age (65.7%; *n* = 65). Transportation is inherently stressful for young calves [38]. During transportation, calves are exposed to multiple stressors, including overcrowding and limited ventilation [39]; increased risk of injury due to limited space allowance [40,41,42,43]; and higher energy demands which may have a detrimental impact on immune function, increasing susceptibility to disease in immunologically naïve neonates [44,45]. The current work thus reflects prevailing concerns among animal welfare scientists regarding the stressors of transport on neonatal calves [39,46,47,48].

A cohort effect was found in veterinary perceptions towards the minimum age of fitness for transport. Female veterinarians supported a higher mean age of fitness for transport than male veterinarians (*p* = 0.021). Females have been found to place greater importance on animal welfare than males [49], with female veterinarians placing consistently more emphasis on how animals are treated [50,51]. Females have also been found to have a stronger belief in an animal’s capacity to experience emotion, such as hunger and distress [28,50,52,53]. The stressors associated with transport are both multiple and cumulative. Given that female veterinarians are more likely to recognize the negative impacts of such stressors on the affective experiences of calves than male veterinarians, this may explain the gender differences observed in the current work.

Veterinarians working in companion animal practice and mixed animal practice supported a higher age of fitness for transport (*p* = 0.001) than veterinarians working in large animal practice. An association between working in large animal practice and reduced concern for farm animal welfare has been reported among veterinary students. A study by Levine and colleagues [54] found that veterinary students aspiring to work in large animal practice perceived certain contentious practices (e.g., hot iron branding) as more acceptable than students intending to work with companion animals. Similarly, Ostovic and colleagues [55] reported that students aspiring to work with companion animals shared greater concern towards farm animal welfare than those that had elected to work with farm animals. This phenomenon has been attributed to emotional detachment among practitioners due to desensitization towards emotion-provoking stimuli [50]. Desensitization has been found to coincide with the time of greatest exposure, perhaps as a mechanism for coping with stressful and ethically challenging situations that are frequently encountered in veterinary practice [50,55,56,57].

Although the maximum time off feed for young calves was reduced from 30 to 24 h [3], veterinarians in the current work strongly disagreed with the amended maximum limit (97.1%; *n* = 101). Further, there was considerable support for reducing the maximum time off feed to 12 h or less (91.3%; *n* = 95), with the greatest proportion of veterinarians advocating for a maximum limit of 6 h (47.1%; *n* = 49). Importantly, the metabolic parameters of neonates are fundamentally different from older calves [40,47], as young calves have higher metabolic demands and lower energy reserves [47,58]. While energy deficiencies can be countered through the mobilization of body reserves [40,41,42], stressors associated with transport may disturb homeostatic mechanisms and place significant constraints on the capacity for young calves to fully compensate for such deficiencies [59]. Given that neonatal calves rely almost entirely on milk for nutritional enrichment, there are significant welfare concerns for calves held off feed and deprived of nutrients for up to 24 h.

Veterinarians working in companion animal practice and mixed animal practice supported a lower time off feed (*p* = 0.005) than veterinarians working in large animal practice. In line with these findings, Mariti and colleagues [60] found that veterinary students who elected to work in large animal practice expressed less concern for the emotional aspects of animal welfare when compared with students who elected to work with companion animals. Given that hunger is among the capabilities of sentient animals [61], there are parallels between perceptions of hunger and perceptions of sentience. Identifying differences in perception between veterinarians working in companion animal practice and veterinarians working in large animal practice is important because it highlights the role that desensitization may play in practice. As previously discussed, veterinarians working in large animal practice are confronted with navigating multiple, often competing, conflicts of interest. While veterinarians are trained to focus on animal health at an individual level, a veterinarian working in large animal practice is often tasked with managing animal health on a collective level [62]. This shift towards collective care places a greater emphasis on the utility or instrumental value of animals, rather than the affective state of individual animals [55]. An enhanced understanding of differences in perceptions may help to inform strategies aimed at emphasizing the psychological welfare of farm animals in veterinary curricula and continuing education.

Under new calf welfare regulations, a 12 h maximum transport duration was introduced. However, veterinarians in the present study firmly disagreed with this limit (99.0%; *n* = 102), with most veterinarians expressing support for a maximum limit of 6 h or less (93.1%; *n* = 95). Studies have found significant effects of haul distance, and therefore transport duration, on increased concentrations of plasma cortisol and noradrenaline indicating a heightened stress response [43], along with increased urea concentrations, β-hydroxybutyric acid levels, and lipid and protein catabolism consistent with a state of energy crisis [41]. Further, higher concentrations of creatine kinase activity from prolonged periods of standing and bracing against movement have been reported [40,41,42]. The negative welfare impacts of prolonged transport are therefore well established [43,46,63,64]. Boulton and colleagues [1] found that for every additional hour of transportation, the risk of calf mortality or condemnation increased. Importantly, the welfare risk was linear, that is, there was no threshold below which the duration of transport would be safe for young calves; rather, shorter transport durations posed lower risk.

These findings support the outcome of public submissions pursuant to the proposed regulatory amendments [3], reflecting prevailing concerns among animal welfare advocates. In line with the current work, submitters strongly disagreed (90%; *n* = 62) with the proposed 4 day minimum age of fitness for transport and the 24 h maximum time off feed for young calves (89%; *n* = 63), and favored an 8 h maximum transport duration (74%; *n* = 50), as opposed to the industry standard of 12 h [3]. Despite strong advocacy to strengthen the welfare protection afforded to young calves, farm industry representatives argued that these changes may lead to significant economic and practical constraints on producers [65]. The consultation process thus raised concerns regarding the asymmetries that exist between industry voices and welfare advocates in a trade-off between protecting the welfare of young calves and safeguarding New Zealand’s economic interests [12,13].

### 4.2. Veterinary Concerns for Calf Welfare Compromise

In the current work, veterinarians expressed concerns for calf welfare across a broad spectrum of issues. The greatest concerns for calf welfare compromise were based on issues relating to calf management, including transportation, inadequate housing, and bobby calf mistreatment. Consistent with the current study, Ventura and colleagues [66] found that the low prioritization of calf management is an important concern among veterinarians. A recent study in New Zealand found that young bobby calves were generally allocated less space in housing and were less likely to be fed colostrum than replacement calves [1]. While veterinary concerns for animal welfare have traditionally concentrated on animal health and functioning [67], the role of the veterinarian has changed over time to encompass the assessment of animal welfare in response to shifts in social expectations [10,62,68]. The concerns of veterinarians in the current work reflect this shift in veterinary ethics, with veterinarians questioning the acceptability of certain calf management practices [15,62,69].

### 4.3. Perceived Barriers to Welfare-Related Change

The likelihood that welfare-related change will be implemented is governed, at least in part, by what barriers may inhibit its implementation [70]. By examining both the qualitative and quantitative data in the current work side-by-side, strong corroboration was found in perceptions towards the most important barriers to welfare-related change, with limited knowledge and costs among the greatest barriers. Concerns for the costs of improving welfare has frequently surfaced [26,68], as welfare-related improvements can induce substantial costs across the production chain [30,31]. With regard to veterinarians’ identification of limited knowledge as a barrier to improving calf welfare on-farm, it is important to consider whether current animal welfare science knowledge is accessible to farmers [71], as this may lead to inaction regarding welfare-related change [26]. The ability for veterinarians to translate animal welfare knowledge into on-farm application is thus an important issue [72]. However, veterinarians in both the current work and previous studies [7,26] have expressed the difficulty of transferring knowledge on the basis that, in doing so, they may risk losing clients. Given that these concerns could have a negative impact on efforts to improve calf management, there is scope for future work on veterinarian–client communication to equip veterinarians with strategies to facilitate improved welfare outcomes in practice.

### 4.4. Limitations

A limitation of the current work is that participation in the survey was voluntary, which may have introduced self-selection bias. For instance, veterinarians working directly with calves are likely to have an invested interest in calf welfare as they are affected by the items in question. For this reason, respondents may not have been representative of the veterinary profession as a whole. While the extent of self-selection bias, if any, is difficult to assess, the demographic data indicated a diverse range of respondents across gender, age, graduation, and species emphasis. Further, the age and gender distribution of respondents in the current work is representative of the higher proportion of females entering the veterinary profession in New Zealand—a trend which is consistent with the VCNZ Workforce Report [73].

The current work was based on veterinary opinions towards current legislation, and given that the current legislative framework pertaining to calf welfare in New Zealand does not differentiate between calves reared for dairy or beef, this study did not make a distinction. Thus, there is future scope to explore whether veterinarians have different concerns for calf welfare depending on whether a calf is reared for dairy or beef, and this merits further investigation.

### 4.5. Implications

As the first study to investigate veterinarians’ perceptions towards calf welfare legislation in New Zealand, the findings raised concerns regarding the level of welfare protection afforded to young calves. Despite recent regulatory amendments introduced to address areas of highest risk to calf welfare, the resulting regulations have been criticized for favoring industry voices, incentivized to support standards that would otherwise fall below the general provisions of the Act [12,13]. In response to the increasing public demand for strengthening farm animal welfare standards [62,68,74], veterinarians have been encouraged to question the accepted norms and practices of calf management [15], which represents an important shift in veterinary ethics. Veterinarians in the current work disagreed with the acceptability of certain calf welfare specifications and advocated for greater protection of young calves in New Zealand, which suggests that veterinarians would likely support strengthening the level of welfare protection afforded to calves.

Given the asymmetries that exist between the current regulatory framework and veterinary perspectives, the findings suggest that more needs to be done to improve calf welfare in New Zealand. The knowledge gained from this study provides scope for researchers to carry out a systematic and independent review of developments in animal welfare science, which would further guide regulatory reform by identifying regulations that fall short of current scientific knowledge. Additionally, the recognition of veterinary concerns for calf welfare and barriers to welfare-related change can be used to inform intervention strategies targeted at improving calf welfare across the production chain. Further, the demographic differences identified in this study suggest that there is a need for a greater emphasis on emerging knowledge in animal welfare science in veterinary curricula and continuing education.

## 5. Conclusions

Despite advancements in animal welfare science, which have led to a greater understanding of the welfare needs of calves, substantive improvements are necessary in order to reconcile New Zealand’s existing regulatory regime with developments in scientific knowledge. The current work revealed considerable support for improving calf management practices, found in veterinarians’ shared affinity for increasing the level of welfare protection afforded to calves in New Zealand. In the current work, veterinarians strongly disagreed with the specifications of certain calf welfare regulations, identified a broad spectrum of concerns for calf welfare compromise across the production chain, and recognized barriers to welfare-related change. Given the opportunity, veterinarians in New Zealand would likely support efforts to strengthen the legal welfare protection afforded to calves in practice and policy.

## Figures and Tables

**Table 1:** Survey items used to determine whether veterinary opinions align with certain welfare provisions pertaining to young calves (up to 14 days of age) in New Zealand.

Question Items	Response Options
In your opinion, what is the acceptable minimum age following birth that young calves are fit for transport?	4 days, 10 days, 14 days, other (please specify)
In your opinion, what is the acceptable maximum time off feed for young calves prior to slaughter?	6 h, 12 h, 24 h, other (please specify)
In your opinion, what is the acceptable maximum duration of transportation for young calves?	6 h, 8 h, 10 h, 12 h, other (please specify)

**Table 2:** Likert-type scale items concerning potential barriers to welfare-related change, adapted from the existing literature.

Scale Items	Adapted from
An increase in costs associated with welfare-related change	[26,29,30,31]
Limited enforceability of animal welfare regulations	[13,26,32]
Practical limitations of monitoring animal welfare	[26,33,34]
Limited animal welfare knowledge among farm workers	[26,35]
Resistance to welfare-related changes among industry stakeholders	[29,35]
Limited funding to support welfare-related change	[26,34,36]

**Table 3:** An overview of the sample demographics.

Demographic Variable	*n*	%
Years since graduation (*N* = 103; $\bar{x}$ = 23.75; σ = 14.23)		
0–10	23	22.2
11–20	21	20.5
21–30	29	28.0
31–40	15	14.7
41 and over	15	14.7
Gender (*N* = 102)		
Male	49	48.0
Female	53	52.0
Age (*N* = 103; $\bar{x}$ = 48.75; σ = 13.92)		
24–30	12	11.6
31–40	21	20.2
41–50	23	22.2
51–60	25	24.1
61 and over	22	21.4
Species emphasis (*N* = 100)		
Large animal practice	41	41.0
Mixed animal practice	37	37.0
Companion animal practice	22	22.0

## Data Availability

The data presented in this study are available on request from the corresponding author. The data are not publicly available due to privacy reasons.

## Appendix A

**Table A1:** Qualitative content analysis of veterinary concerns regarding the areas at highest risk of welfare compromise in calves using a weighted rank score (WRS).

Code	Examples	Rank 1	Rank 2	Rank 3	Total	WRS ^a^
Disbudding	“Disbudding”; “Dehorning”	11	10	8	29	**61**
Castration	“Castration”; “Castration without analgesia”	4	5	-	9	**22**
Supernumerary teat removal	“Extra teat removal”	-	-	2	2	**2**
Ear tagging	“Ear tagging causes pain in calves”	-	1	-	1	**2**
Gastrointestinal disease	“Scours”; “Infectious scours”	3	4	4	11	**21**
Infection (unspecified)	“Disease”	-	2	3	5	**7**
Dystocia	“Survival at birth”	2	-	-	2	**6**
Transportation	“Trucking young calves to slaughter”; “Poor transport practices”	15	16	8	39	**85**
Inadequate housing	“Inadequate environments”; “Exposure to harsh conditions”	8	16	10	34	**66**
Mistreatment of “bobby” calves	“Treatment of bobby calves”; “Abuse of bobby calves”	16	4	4	24	**60**
Malnutrition	“Lack of food and water”; “Inadequate colostrum”	9	6	16	31	**55**
Inadequate pain management	“Lack of local anesthesia and analgesia”	5	11	7	23	**44**
Poor husbandry management	“On=farm management”; “Poor animal husbandry”	5	7	6	18	**35**
Maternal separation	“Age of removal from mother”; “Distress from removal from mother”	6	1	3	10	**23**
Limited knowledge	“Lack of training”; “Uneducated, untrained, and ill-equipped”	5	1	3	9	**20**
Stressful handling	“Poor handling”	2	4	6	12	**20**
Poor hygiene practices	“Disease control and hygiene practices”	1	4	1	6	**12**
Poor slaughter practices	“Slaughter methods”	2	1	2	5	**10**
Inadequate legislation	“New regs still not fully protecting calves”	1	1	-	3	**8**

^a^ The WRS was calculated to take into account the frequency of the code and its weighted rank to ensure that the greatest concerns had a proportional representation during analysis.

## Appendix B

**Table A2:** Qualitative content analysis of veterinary perceptions concerning barriers to welfare-related change using a weighted rank score (WRS).

Code	Examples	Rank 1	Rank 2	Rank 3	Total	WRS ^a^
Inadequate legislation	“Lack of welfare laws”; “Lack of monitoring of welfare”	4	9	13	26	**43**
Ingrained practices	“Accommodation to existing practice”; “Old-school farming”; “Tradition”	19	14	4	37	**89**
Resistance to change	“Resistance to changing current practice”; “Social inertia”	14	8	7	29	**65**
Lack of empathy	“Lack of concern”; “Lack of empathy among farmers”	4	5	3	12	**25**
Costs	“Price of analgesics”; “Costs of implementing change”	21	29	18	68	**139**
Practical limitations	“Increased labor demand”; “Time constraints”	7	8	11	26	**48**
Poor management	“Poor farming practices”; “Negligence”	3	-	4	7	**13**
Veterinarian communication	“Lack of courage among vets”	2	1	3	6	**10**
Limited knowledge	“Ignorance of poor welfare’; ‘Lack of education’	24	19	18	68	**139**

^a^ The WRS was calculated to take into account the frequency of the code and its weighted rank to ensure that the greatest concerns had a proportional representation during analysis.

## References

[1] Boulton A., Kells N., Beausoleil N., Cogger N., Johnson C., Palmer A., Laven R., O’Connor C., Webster J. (2018). Bobby Calf Welfare across the Supply Chain—Final Report for Year 1.

[2] Statistics New Zealand. http://nzdotstat.stats.govt.nz/wbos/index.aspx?datasetcode=tablecode7423.

[3] Ministry for Primary Industries (2017). Animal Welfare Regulations: Summary Report on Public Consultation April/May 2016.

[4] Mellor D. (2000). Bobby calf welfare. Surveillance.

[5] Mee J.F. (2013). Why Do So Many Calves Die on Modern Dairy Farms and What Can We Do about Calf Welfare in the Future?. Animals.

[6] Rodriguez Ferrere M.B., King M., Larsen L.M. (2019). Animal Welfare in New Zealand: Oversight, Compliance and Enforcement.

[7] Sumner C.L. (2018). Promoting Farmer and Veterinarian Cooperation to Improve Dairy Calf Welfare. Ph.D. Thesis.

[8] Cuttance E.L., Mason W.A., Laven R.A., McDermott J., Phyn C. (2017). Prevalence and calf-level risk factors for failure of passive transfer in dairy calves in New Zealand. N. Z. Vet. J..

[9] Renaud D.L., Duffield T.F., LeBlanc S.J., Haley D.B., Kelton D.F. (2017). Management practices for male calves on Canadian dairy farms. J. Dairy Sci..

[10] Weary D.M., von Keyserlingk M.A.G. (2017). Public concerns about dairy-cow welfare: How should the industry respond?. Anim. Prod. Sci..

[11] Farmwatch. https://vimeo.com/146749967.

[12] Morris M.C. (2011). The Use of Animals in New Zealand: Regulation and Practice. Soc. Anim..

[13] Rodriguez Ferrere M.B. (2018). Codes vs. regulations: How best to enforce animal welfare in New Zealand?. Altern. Law J..

[14] New Zealand Veterinary Association (2016). NZVA position on Bobby Calf Welfare. Dairy Cattle Vet. Newsl..

[15] New Zealand Veterinary Association. https://www.nzva.org.nz/news/407325/NZVA-statement-The-Animal-Welfare-Act-must-protect-animals.htm.

[16] Derks M., van Werven T., Hogeveen H., Kremer W.D.J. (2013). Veterinary herd health management programs on dairy farms in the Netherlands: Use, execution, and relations to farmer characteristics. J. Dairy Sci..

[17] Pothmann H., Nechanitzky K., Sturmlechner F., Drillich M. (2014). Consultancy to dairy farmers relating to animal health and herd health management on small- and medium-sized farms. J. Dairy Sci..

[18] Kauppinen T., Vainio A., Valros A., Rita H., Vesala K.M. (2010). Improving animal welfare: Qualitative and quantitative methodology in the study of farmers’ attitudes. Anim. Welf..

[19] Winder C.B., LeBlanc S.J., Haley D.B., Lissemore K.D., Godkin M.A., Duffield T.F. (2016). Practices for the disbudding and dehorning of dairy calves by veterinarians and dairy producers in Ontario, Canada. J. Dairy Sci..

[20] Wolf C.A., Tonsor G.T., McKendree G.S., Thomson D.U., Swanson J.C. (2016). Public and farmer perceptions of dairy cattle welfare in the United States. J. Dairy Sci..

[21] Laven R.A., Huxley J.N., Whay H.R., Stafford K.J. (2009). Results of a survey of attitudes of dairy veterinarians in New Zealand regarding painful procedures and conditions in cattle. N. Z. Vet. J..

[22] Fajt V.R., Wagner S.A., Norby B. (2011). Analgesic drug administration and attitudes about analgesia in cattle among bovine practitioners in the United States. J. Am. Vet. Med..

[23] Hewson C.J., Dohoo I.R., Lemke K.A., Barkema H.W. (2007). Factors affecting Canadian veterinarians use of analgesics when dehorning beef and dairy calves. Can. Vet. J..

[24] Huxley J.N., Whay H.R. (2006). Current attitudes of cattle practitioners to pain and the use of analgesics in cattle. Vet. Rec..

[25] Bracke M.B.M., Edwards S.A., Engel B., Buist W.G., Algers B. (2008). Expert opinion as ‘validation’ of risk assessment applied to calf welfare. Acta Vet. Scand..

[26] Ventura B.A., Weary D.M., Giovanetti A.S., von Keyserlingk M.A.G. (2016). Veterinary perspectives on cattle welfare challenges and solutions. Livest. Sci..

[27] Lund V., Coleman G., Gunnarsson S., Appleby M.C., Karkinen K. (2006). Animal welfare Science—Working at the interface between the natural and social sciences. Appl. Anim. Behav. Sci..

[28] Thompson C.S. (2016). 2016 Assessing Attitudes towards Welfare and Pain in Farm Animals. Ph.D. Thesis.

[29] Bock B.B., van Huik M.M. (2007). Animal welfare: The attitudes and behaviour of European pig farmers. Br. Food J..

[30] Bracke M.B.M., Greef K.H.D., Hopster H. (2005). Qualitative stakeholder analysis for the development of sustainable monitoring systems for farm animal welfare. J. Agric. Environ. Ethics.

[31] Grethe H. (2017). The Economics of Farm Animal Welfare. Annu. Rev. Resour. Econ..

[32] White S. (2013). Into the Void: International Law and the Protection of Animal Welfare. Glob. Policy.

[33] Barnett J.L., Hemsworth P.H. (2009). Welfare Monitoring Schemes: Using Research to Safeguard Welfare of Animals on the Farm. J. Appl. Anim. Welf. Sci..

[34] Whay H.R. (2007). The journey to animal welfare improvement. Anim. Welf..

[35] Te Velde H., Aarts N., Van Woerkum C. (2002). Dealing with ambivalence: Farmers’ and consumers’ perceptions of animal welfare in livestock breeding. J. Agric. Environ. Ethics.

[36] Millman S.T., Duncan I.J.H., Stauffacher M., Stookey J.M. (2004). The impact of applied ethologists and the International Society for Applied Ethology in improving animal welfare. Appl. Anim. Behav. Sci..

[37] Dillman D.A. (2000). Mail and Internet Surveys: The Tailored Design Method.

[38] Schwartzkopf-Genswein K.S., Booth-McLean M.E., Shah M.A., Entz T., Bach S.J., Mears G.J., Schaefer A.L., Cook N., Church J., McAllister T.A. (2007). Effects of pre-haul management and transport duration on beef calf performance and welfare. Appl. Anim. Behav. Sci..

[39] Goldhawk C., Janzen E., González L.A., Crowe T., Kastelic J., Pajor E., Schwartzkopf-Genswein K.S. (2014). Trailer microclimate and calf welfare during fall-run transportation of beef calves in Alberta. J. Anim. Sci..

[40] Jongman E.C., Butler K.L. (2014). The Effect of Age, Stocking Density and Flooring during Transport on Welfare of Young Dairy Calves in Australia. Animals.

[41] Stafford K.J., Mellor D.J., Todd S.E., Gregory N.G., Bruce R.A., Ward R.N. (2001). The physical state and plasma biochemical profile of young calves on arrival at a slaughter plant. N. Z. Vet. J..

[42] Todd S.E., Mellor D.J., Stafford K.J., Gregor N.G., Bruce R.A., Ward R.N. (2000). Effects of food withdrawal and transport on 5- to 10-day-old calves. Res. Vet. Sci..

[43] Uetake K., Tanaka T., Sata S. (2011). Effects of haul distance and stocking density on young suckling calves transported in Japan. Anim. Sci. J..

[44] Earley B., Buckham Sporer K., Gupta S. (2017). Invited review: Relationship between cattle transport, immunity and respiratory disease. Animal.

[45] Riondato F., D’Angelo A., Miniscalco B., Bellino C., Guglielmino R. (2008). Effects of road transportation on lymphocyte subsets in calves. Vet. J..

[46] Cave J.G., Callinan A.P.L., Woonton W.K. (2005). Mortalities in bobby calves associated with long distance transport. Aust. Vet. J..

[47] Fisher A.D., Colditz I.G., Lee C.F. (2009). The influence of land transport on animal welfare in extensive farming systems. J. Vet. Behav..

[48] Nielsen B.L., Dybkjær L., Herskin M.S. (2011). Road transport of farm animals: Effects of journey duration on animal welfare. Animal.

[49] Cornish A.R., Caspar G.L., Collins T., Degeling C., Fawcett A., Fisher A.D., Freire R., Hazel S.J., Jennifer H., Johnson A.J. (2016). Career Preferences and Opinions on Animal Welfare and Ethics: A Survey of Veterinary Students in Australia and New Zealand. J. Vet. Med. Educ..

[50] Paul E.S., Podberscek A.L. (2000). Veterinary education and students’ attitudes towards animal welfare. Vet. Rec..

[51] Sabuncuoglu N., Coban O. (2008). Attitudes of Turkish veterinarians towards animal welfare. Anim. Welf..

[52] Ellingsen K., Zanella A.J., Bjerkås E., Indrebø A. (2010). The Relationship between Empathy, Perception of Pain and Attitudes toward Pets among Norwegian Dog Owners. Anthrozoös.

[53] Knight S., Vrij A., Cherryman J., Nunkoosing K. (2004). Attitudes toward animal use and belief in animal mind. Anthrozoös.

[54] Levine E.D., Mills D.S., Houpt K.A. (2005). Attitudes of Veterinary Students at One US College toward Factors Relating to Farm Animal Welfare. Anim. Welf..

[55] Ostovic M., Mikus T., Pavicic Z., Matkovic K., Mesic Z. (2017). Influence of socio-demographic and experiential factors on the attitudes of Croatian veterinary students towards farm animal welfare. Vet. Med..

[56] Batchelor C.E.M., McKeegan D.E.F. (2012). Survey of the frequency and perceived stressfulness of ethical dilemmas encountered in UK veterinary practice. Vet. Rec..

[57] Pollard-Williams S., Doyle R.E., Freire R. (2014). The influence of workplace learning on attitudes toward animal welfare in veterinary students. J. Vet. Medic. Educ..

[58] Stull C., Reynolds J. (2008). Calf Welfare. Vet. Clin. N. Am. Food Anim..

[59] Adenkola A.Y., Ayo J.O. (2010). Physiological and behavioural responses of livestock to road transportation stress: A review. Afr. J. Biotechnol..

[60] Mariti C., Pirrone F., Albertini M., Gazzano A., Diverio S. (2018). Familiarity and Interest in Working with Livestock Decreases the Odds of Having Positive Attitudes towards Non-Human Animals and Their Welfare among Veterinary Students in Italy. Animals.

[61] Broom D.M., Broom D.M. (2010). The Qualities That Make Up Sentience. Sentience and Animal Welfare.

[62] Meijboom F.L. (2018). More than just a vet? Professional integrity as an answer to the ethical challenges facing veterinarians in animal food production. Food Ethics.

[63] Grigor P.N., Cockram M.S., Steele W.B., Le Sueur C.J., Forsyth R.E., Guthrie J.A., Johnson A.K., Sandilands V., Reid H.W., Sinclair C. (2001). Effects of space allowance during transport and duration of mid-journey lairage period on the physiological, behavioural and immunological responses of young calves during and after transport. Anim. Sci..

[64] Knowles T.G., Warriss P.D., Brown S.N., Edwards J.E., Watkins P.E., Phillips A.J. (1997). Effects on calves less than one month old of feeding or not feeding them during road transport of up to 24 hours. Vet. Rec..

[65] Ministry for Primary Industries (2016). Proposed Animal Welfare Regulations—Young Calves and Live Animal Exports.

[66] Ventura B.A., von Keyserlingk M.A.G., Weary D.M. (2015). Animal welfare concerns and values of stakeholders within the dairy industry. J. Agric. Environ. Ethics.

[67] Fraser D. (2009). Assessing Animal Welfare: Different Philosophies, Different Scientific Approaches. Zoo Biol..

[68] Vanhonacker F., Verbeke W. (2014). Public and Consumer Policies for Higher Welfare Food Products: Challenges and Opportunities. J. Agric. Environ. Ethics.

[69] Hernandez E., Fawcett A., Brouwer E., Rau J., Turner P.V. (2018). Speaking Up: Veterinary Ethical Responsibilities and Animal Welfare Issues in Everyday Practice. Animals.

[70] Rosenstock I.M. (1974). The Health Belief Model and preventive health behaviour. Health Educ. Monogr..

[71] Bran J.A., Daros R.R., von Keyserlingk M.A.G., Hötzel M.J. (2018). Lameness on Brazilian pasture based dairies—Part 1: Farmers’ awareness and actions. Prev. Vet. Med..

[72] Kristensen E., Jakobsen E.B. (2011). Challenging the myth of the irrational dairy farmer; understanding decision-making related to herd health. N. Z. Vet. J..

[73] Veterinary Council of New Zealand (2018). The New Zealand Veterinary Workforce in 2017–2018.

[74] Mellor D.J., Webster J.R. (2014). Development of animal welfare understanding drives change in minimum welfare standards. Rev. Sci. Tech..

